# Dynamic motion trajectory control with nanoradian accuracy for multi-element X-ray optical systems via laser interferometry

**DOI:** 10.1038/s41377-025-01774-5

**Published:** 2025-03-20

**Authors:** Sina M. Koehlenbeck, Lance Lee, Mario D. Balcazar, Ying Chen, Vincent Esposito, Jerry Hastings, Matthias C. Hoffmann, Zhirong Huang, May-Ling Ng, Saxon Price, Takahiro Sato, Matthew Seaberg, Yanwen Sun, Adam White, Lin Zhang, Brian Lantz, Diling Zhu

**Affiliations:** 1 Edward L. Ginzton Laboratory, Stanford University, Stanford, CA 94305 USA; 2 Linac Coherent Light Source, SLAC National Accelerator Laboratory, Stanford, CA 94309 USA; 3 Department of Applied Physics, Stanford University, Stanford, CA 94305 USA

**Keywords:** X-rays, Optical sensors

## Abstract

The past decades have witnessed the development of new X-ray beam sources with brightness growing at a rate surpassing Moore’s law. Current and upcoming diffraction limited and fully coherent X-ray beam sources, including multi-bend achromat based synchrotron sources and high repetition rate X-ray free electron lasers, puts increasingly stringent requirements on stability and accuracy of X-ray optics systems. Parasitic motion errors at sub-micro radian scale in beam transport and beam conditioning optics can lead to significant loss of coherence and brightness delivered from source to experiment. To address this challenge, we incorporated optical metrology based on interferometric length and angle sensing and real-time correction as part of the X-ray optics motion control system. A prototype X-ray optics system was constructed following the optical layout of a tunable X-ray cavity. On-line interferometric metrology enabled dynamical feedback to a motion control system to track and compensate for motion errors. The system achieved sub-microradian scale performance, as multiple optical elements are synchronously and continuously adjusted. This first proof of principle measurement demonstrated both the potential and necessity of incorporating optical metrology as part of the motion control architecture for large scale X-ray optical systems such as monochromators, delay lines, and in particular, X-ray cavity systems to enable the next generation cavity-based X-ray free electron lasers.

## Abstract (web summary)

Future cavity-based XFEL sources require optics stable to hundreds of nanometers and nanoradian. We present how laser interferometry metrology and feedback control achieve this even during energy and delay scans.

## Graphical abstract

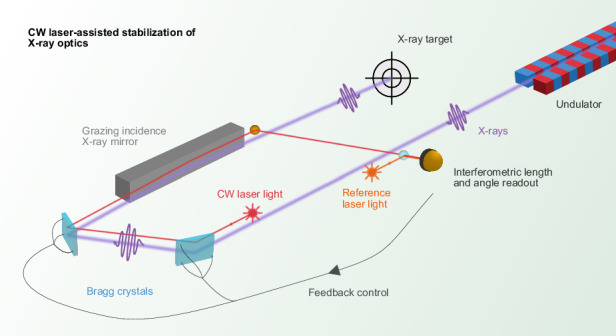

## Introduction

Modern X-ray beam sources and their applications rely heavily on high performance precision motion control mechanisms. The spatial motion of the X-ray beam transversal to the target sample determines the spatial resolution for imaging, while longitudinal displacements and the temporal stability of the pulse sequence determine the temporal resolution of the time-resolved measurements. X-ray microscopes, for example, now routinely produce tomographs with 10 nm spatial resolution in 3D on buried nano structures^1^. Precision nano focusing optics were able to produce highly focused X-ray radiation from free electron lasers (FELs) to generate intensities of 10^20^ W cm^−2^, demonstrating nonlinear optical effects such as two-photon absorption and atomic-shell FELs^2–4^. However, our current motion control capabilities for multi-optic systems result in pointing jitter and misalignment that often exceeds the intrinsic angular divergence of the X-ray sources, which in turn effectively reduces the brightness and coherence^5–9^. One example for this is the key beam transport and conditioning optic, the X-ray monochromator. It usually consists of multiple optical elements and requires high accuracy coordinated motions to maintain the target beam position at tens to hundreds of meters downstream. Angular deviation from the ideal motion trajectory at a small fraction of the beam divergence scale leads to unacceptable beam motion at the sample position. This condition must not only be met for a static alignment of the individual optics, but also during an energy scan, which sometimes requires up to meter-long linear translation of the crystal optics while needing the parasitic angular motion to be well below a 100 nrad scale^10,11^.

High-precision beam path stability will also be required in realizing X-ray regenerative amplifiers. X-ray generation in cavity-based amplifier systems promises to improve longitudinal coherence and beam brightness by 2–3 orders of magnitude compared to current and planned single-pass self-amplified spontaneous emission (SASE) X-ray FEL sources^12–15^. Pioneering experiments demonstrate key technologies such as multi-pass circulation of X-rays^16^ and prototype facilities are on their way to demonstrate the multi-pass amplification of X-ray laser radiation^17,18^. Stable X-ray cavities are required to achieve the necessary superposition of the recirculating X-ray pulses with the gain medium, i.e., the relativistic electron bunch in the undulator. For nonlinear X-ray spectroscopy, X-ray quantum optics, and quantum metrology it is furthermore necessary to adjust cavity resonance wavelengths. Such a tunable cavity-based X-ray laser source must support multiple optical elements moving synchronously in coordination, while maintaining the overall cavity length and angular alignment^19^.

**Fig. 1:**
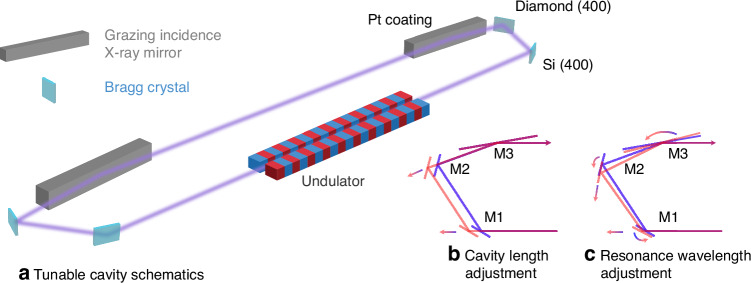
Schematic drawing of a cavity-based regenerative X-ray amplifier and detailed diagrams for adjusting the cavity length and the resonance wavelength. **a** Schematics of a six optic X-ray cavity optics layout that provides a small range of tunability. **b** Arrows indicating coordinated motion required for M1 and M2 to extend the cavity length by going from the blue to the red beam path, with a fixed input and output beam path. **c** Arrows indicating coordinate motion required for M1, M2, and M3, to adjust the cavity resonance wavelength, and hence the photon energy of the reflected X-rays, while maintaining the overall cavity length as well as output beam path

One of the tunable cavity geometries under consideration is shown in Fig. 1, which would be compatible with the planned linac coherent light source (LCLS)-II HE undulator infrastructure^9^. Two symmetric groups of optics are laid out at the two opposite ends of a series of undulators. The downstream optics group introduces a 180° turn to the X-ray radiation with a relatively small offset, thus limiting the transverse footprint of the optical layout to be compatible with the undulator tunnel layout. The upstream optics group creates another U turn and send the X-ray beam back into the undulators, to seed the generation of more coherent X-ray laser light. The pair of Bragg optics can be chosen to fit the desired photon energy with a combined Bragg angle value just below 90°, while the grazing incidence mirror completes the precise 180° beam return. The acceptance angle of the mirror in grazing incident covers the angular positions required to continuously adjust the photon energy of several tens of eV with the two crystals reflecting by Bragg resonance. Such a cavity has two important adjustable degrees of freedom, which are shown in Fig. 1b, c: cavity length, and cavity resonance wavelength. The cavity length must be scanned and set to match the repetition rate of the electron bunch generation, while the cavity resonance wavelength needs to be matched to the desired X-ray wavelength, and hence photon energy, for the sample under test.

The motion accuracy requirement of the cavity optics is set by the X-ray pulse transverse size and length, as well as the undulator length. To maintain resonant amplification of the recirculating X-ray pulses, they must overlap with similarly sized electron bunches. In a study by Marcus et al.^14^, two cases were mentioned: the X-ray free-electron laser oscillator (XFELO) with relatively low gain for each pass and a system with higher gain per pass termed the X-ray regenerative amplifier free-electron laser (XRAFEL). Both concepts have their advantages and disadvantages and are aimed at different applications. The XFELO must have low losses and a low extraction rate per pass to generate Fourier-limited, narrow-band (meV) X-ray pulses, for high-resolution spectroscopy. The electron bunch must be stretched in the longitudinal direction to envelop the X-ray pulse. As a result, the electrons are distributed over a large volume and the charge density is reduced, resulting in a weak gain per pass and hundreds of passes until saturation is reached. The requirements for optics stability of the resonator can be calculated analytically from geometric considerations. The tolerable angular mirror motion is quoted to be as low as 10 nrad and 55 nrad^18,19^. In contrast, a XRAFEL generates ultra-short (femtosecond), broadband X-ray pulses, to study ultra-fast processes. The current density in the electron bunch is significantly higher and the transverse overlap of the circulating X-ray pulse with the electron bunch has a resulting lower requirement. The X-ray pulses and electron bunches only need to partially overlap at the beginning of the undulator, because the transverse current density gradient is focusing the X-ray beam onto the electron trajectory. Simulation results show that the calculated RMS (root mean square) angular error tolerance of the mirror can be reduced from *σ*_*m*_ ∼70 nrad to *σ*_*m*_ = 0.35 μrad^14^.

The requirements for the angular and length stability of the regenerative amplifier are difficult to fulfill if the optics are only aligned to the resonance state of the X-rays without feedback control. Even if the optics are well connected to the ground, the necessary maneuvers for a wavelength change can only be realized by a physical movement, which in turn causes undesirable deviations from the nominal position due to parasitic motion errors. In addition, the X-ray pulses are about 10 μm long and the length of the cavity must be maintained to within ~1 μm. The ground movement over several hundred meters of cavity length caused by the Earth tides alone is in the hundredths of a nanometer range^20^. A constant measurement of the alignment and length drift in combination with an active feedback system to control the optics positions is an excellent solution to compensate for this.

In this work we present a motion control architecture, implemented on a three-element X-ray optical configuration that forms half of a tunable X-ray cavity, with the goal of meeting the motion requirements for regenerative amplification of X-ray pulses. An active feedback control system continuously corrects unwanted position deviations by sending laser interferometer signals to steerable optics holders. Laser interferometry has developed into a reliable tool for precision displacement measurements in length and angle^21^. We show how laser interferometry can be used as a guidance system for X-ray optics by continuously monitoring the phase change of a continuous wave IR laser source. The angular tilt and length changes of the optical path are measured with heterodyne interferometry and differential wavefront sensing^22,23^.

**Fig. 2:**
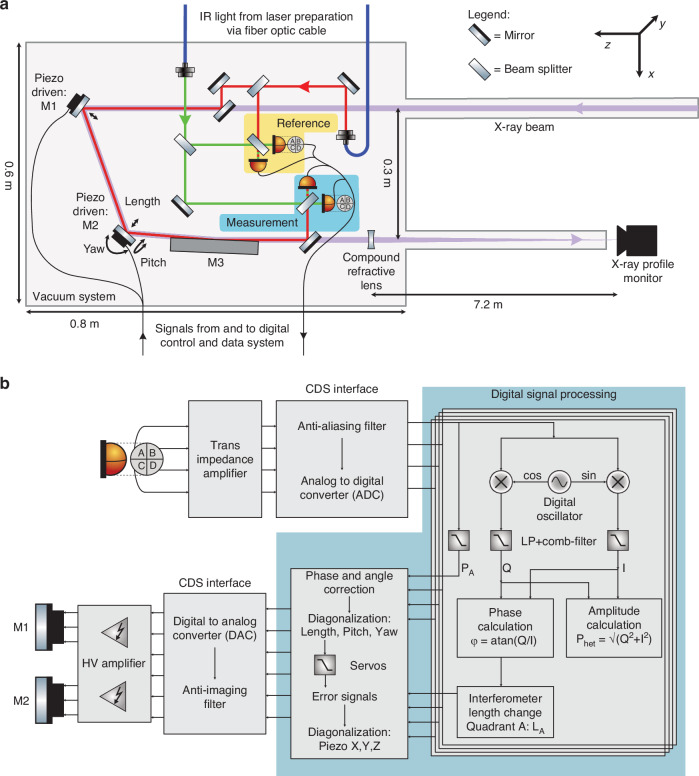
Design of the laser-assisted measurement and feedback system. **a** Layout of the X-ray beam path (purple) and the IR laser optical guidance system (red and green). The X-ray beam is reflected by the optics M1–M3 at a height 25 mm below the optical guide beam and passes the other IR optics through apertures in their posts. M1 and M2 each consist of a Bragg reflector for the X-ray beam and a mirror for the IR laser beam. For each X-ray/IR optics pair, the respective optics are mounted on top of each other within a rigid mechanical holder, mounted on a 3-axis piezo steering stage. M3 reflects both the X-ray and IR wavelength. The guidance system laser beams have a frequency offset of 4096 Hz from each other and their interference generates a beat note at this frequency. Two interferometers are formed from them, and their detection is highlighted in yellow (reference interferometer) and blue (measurement interferometer) shapes. The reference interferometer signal is used to subtract common mode noise from the measurement signal. The compound refractive lens focuses the X-rays onto the profile monitor for beam pointing measurements. **b** Schematics of the data acquisition and control flow for the interferometer readout and motion control feedback architecture. All photodiode elements are first converted to voltages with transimpedance amplifiers (TIAs) and digitized with an ADC. The signal processing is performed with a LIGO style Control and Data System (CDS). The length changes in the interferometer are measured via phase tracking to a digital oscillator at the beat note frequency and control signals computed and distributed to the piezoelectric mirror mounts

The experimental setup of the three-element half X-ray cavity was constructed as shown in Fig. 1b, c. Figure 2 shows the integration of the X-ray optics together with the laser optics for the interferometric measurement. The X-ray optical path is defined by two Bragg crystal reflections and a shallow-angle reflection from a platinum coated X-ray mirror. The Bragg reflections of Si (400) and diamond (400) were chosen such that the cavity resonant wavelength is centered around 8.34 keV, near the Ni K edge. The silicon (400) reflection has a Bragg angle of ~33.2°, and the Diamond (400) reflection ~56.4°. The grazing incidence flat mirror is 300 mm long, with a critical angle of 0.5° at the chosen photon energy. Set to ~0.4° incidence angle it makes up the difference to 90° total incidence angle from the three reflections, thus completes the 180° return of the X-ray beam with a horizontal offset of 300 mm. This mimics half of the tunable cavity as shown in Fig. 1a. Figure 1b illustrates the simultaneous linear motions required for cavity length adjustment. To mimic photon-energy adjustment, the Bragg condition of the two X-ray crystals must be adjusted simultaneously. The grazing incident mirror will change its angle to maintain the incidence angle sum of exactly 90° and maintain the direction of the output beam. In addition, the exit beam offset, and effective path length shall remain constant via translational motions. In total, three mirrors’ rotations plus translations of the two Bragg crystals are needed. This six-axis motion is illustrated in Fig. 1c and needs to be executed synchronously.

The coordinated motion of the energy scan and the cavity length scan are performed with three stacks of stepper motor driven linear and rotation stages. The rotational degrees of freedom are aligned to the center of the X-ray optics rotation point (*θ*: rotation around the *y*-axis), while the linear stages are aligned to the cartesian coordinate system of the experiment (*x*-axis: transverse horizontal, *y*-axis: vertical, *z*-axis: longitudinal horizontal). The absolute translations and rotations per coordinated scan are listed in Table 1.

**Table 1:** Motion trajectory of the energy and delay scan

	Δ*z*_1_ (mm)	Δ*z*_2_ (mm)	Δ*x*_2_ (mm)	Δ*θ*_1_ (deg)	Δ*θ*_2_ (deg)	Δ*θ*_3_ (deg)	Δ*E* (eV)	Δ*t* (ps)
Energy scan	−0.677	−0.600	1.375	0.042	0.182	0.140	10	–
Delay scan	1.499	1.493	−0.013	0.000	0.000	0.000	–	10

The length scan range is equivalent to a round-trip time adjustment of 10 ps. The energy scan covers a range of 10 eV. The motion trajectory for all three optics is calculated to maintain the outgoing beam pointing angles. Due to the small relative range, a linear approximation of the calculated motion trajectory is sufficient. For the energy scan, the path length should be held constant. The expected parasitic motion error from the mechanical stages will lead to changes to outgoing beam’s position and pointing angle. This is measured using an X-ray profile monitor 7.2 m from the X-ray focusing lens, shown in Fig. 2. It images the ‘returned’ X-ray beam profile for each X-ray pulse at 120 Hz. The X-ray beam is focused on the detector scintillator screen with a compound refractive beryllium lens stack just downstream of the grazing incident X-ray mirror. The measurement of the beam position in the focal plane of the compound refractive lens^24^ is primarily sensitive to the beam pointing angle, while it is much less sensitive to the transverse movement of the beam. The angular resolution of the X-ray monitor is about 70 nrad per pixel and results from the measured effective pixel size in the detector plane of 0.51 μm and the distance of the profile monitor to the lens of 7.2 m. The X-ray profile monitor has a pixel size of 3.45 μm, a scintillator, and an imaging optic with a magnification of approximately 6.8 improving the resolution to an effective pixel size of 0.51 μm. The recorded single-pulse X-ray images are fitted with a 2D Gaussian distribution so that the center can be determined to within a tenth of a pixel, or about 50 nm, resulting in a measurement accuracy of ~7 nrad, well below the observed pointing motion.

Parallel to the X-ray beam, offset in the vertical by 25 mm direction an IR laser beam probes the same mechanical mounts, M1–M3, as the X-ray beam and is used to measure the integrated angular errors during length and energy adjustment via heterodyne laser interferometry. The layout of the measurement interferometer is shown in Fig. 2a, with its readout highlighted in the blue shape. The laser interferometer measures the variances in length, pitch, and yaw continually. It is routed as close as possible to the X-ray optical element to increase the correlations between the X-ray and laser beam motion. Unintended length and angle changes introduced by the motion trajectories are compensated for by moving M1 and M2, each mounted to 3-axis piezo steering mirror mount. Length changes are corrected for by simultaneously moving M1 and M2, while deviations from the nominal beam pitch and yaw angles are compensated for by tilting M2. A reference interferometer is shown in the yellow shape, which measures length changes that are unrelated to motions of M1–M3 and are removed by subtraction and feedback control to a fiber stretcher.

The interferometer is in a heterodyne Mach–Zehnder configuration, with a heterodyne frequency offset of 4096 Hz. A detailed description can be found in the methods section and follows the design ideas of the laser interferometer space antenna (LISA) pathfinder metrology^23^ and related experiments^25^. The signal processing chain is sketched in Fig. 2b. The laser interferometer signals are measured with a single and quadrant Si-photodetectors and digitized with a Control and Data System (CDS)^26^ developed for and by the advanced laser interferometer gravitational-wave observatory (aLIGO)^27^ collaboration. In the digital domain the interferometer signals are demodulated at the heterodyne beat frequency and the phase shift over time of this beat note is measured relative to a digital reference sinusoidal signal at the same frequency. The phase shift is converted to a length change as λ/2π, where λ is the laser wavelength of 1064 nm.

Pitch and yaw are measured from the differential phase shift on a quadrant photodiode (QPD), which is referred to as differential wavefront sensing (DWS). However, the radius of curvature of the wavefronts is finite, and therefore a change in position of the laser beams on the detector appears as a phase change in the DWS measurement^28^. The position of the beam on the QPD is well known from a simultaneous differential power sensing (DPS) measurement and can simply be subtracted, making the resulting DWS signal a pure tilt measurement. The corresponding equations can be found in the methods section. The measured interferometer readout jitter with static optics was measured to have a standard deviation of 14 nm in longitude, 72 nrad in pitch and 21 nrad in yaw. We attribute this to the jitter of the optics, as the readout noise limit of the interferometer is estimated to be much lower and the pitch and yaw angles would need to have the same noise level.

From the signals of the interferometer the feedback signals are calculated in real time and converted to analog voltages. They are amplified with a HV amplifier and drive the two three-axis Piezo actuated optics holder. The unity gain frequencies for length and angle control loops are in the order of 10 Hz. The steering degrees of freedom of the Piezo mirror assemblies have been diagonalized to the degrees of freedom, length, pitch, and yaw, of the X-ray beam trajectory, with the help of a second interferometer standing in place of the X-ray beam before the experiment. The control system was originally designed to control 3 degrees of freedom but was extended to 5 degrees of freedom to achieve a further reduction in movement by including translational degrees of freedom during the experiment.

## Results

The setup was installed, and the measurements performed at the X-ray Pump Probe instrument at the Linac Coherence Light Source^29^. The performance of the system is evaluated by direct measurement of the transverse X-ray beam position at the profile monitor. With the control feedback loop off, and all motion axes static, a shot-to-shot variance of the X-ray beam position indicated angular RMS pointing jitter of 0.458 (±0.003) μrad in pitch and 0.169 (±0.001) μrad in yaw, deduced based on the 7.2 m baseline distance of the X-ray focusing lens. By placing the X-ray profile monitor in the focus of the lens, the translations of the X-ray beam on the lens are suppressed at the position of the X-ray profile monitor and the measured jitters primarily are pointing errors. This sets the noise floor for the X-ray measurement.

**Fig. 3:**
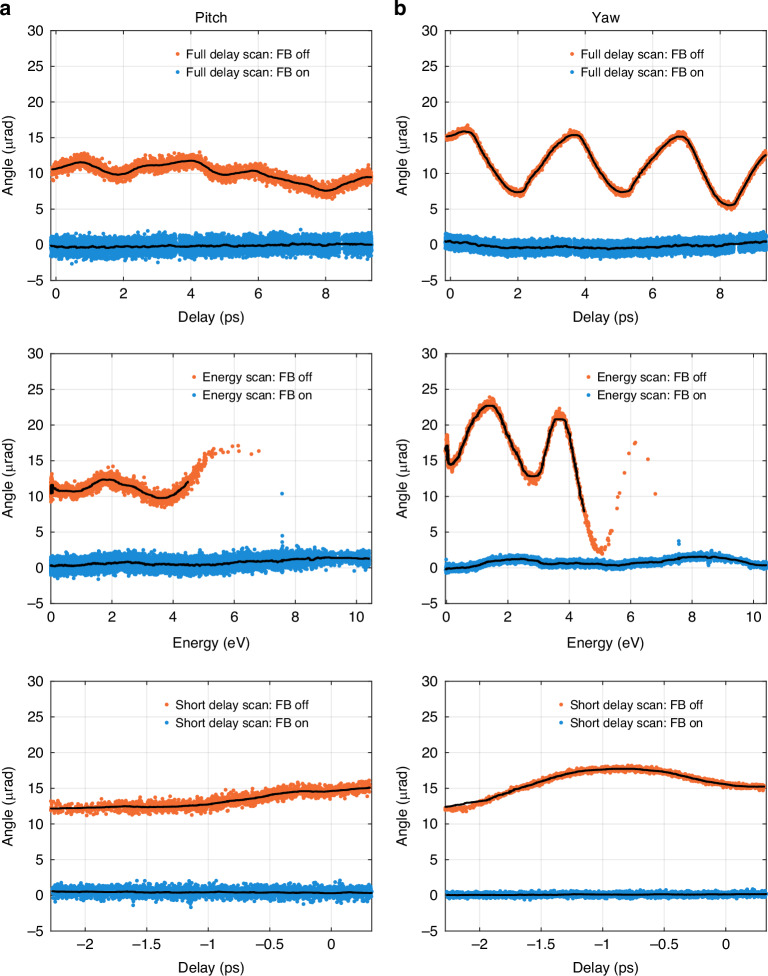
Impact of active motion stabilization to X-ray beam jitter and drift. Shown are the measured center of the X-ray beam on the X-ray profile monitor in units of equivalent beam tilt. The red data points were taken during delay and energy scans without feedback control and blue show the equivalent scans with the feedback enabled. Panel (**a**) shows pitch (vertical tilt), and (**b**) yaw (horizontal tilt) during a short and long delay scan and an energy scan of the half cavity experiment. The black lines are the moving median average of 180 data points. A reduction in peak-to-peak motion once the feedback loops are active can be seen

The benefit of the active stabilization can be seen in Fig. 3. It shows how the beam pointing errors during two types of delay scans and one energy scan are reduced with the feedback enabled for both pitch (a) and yaw (b). The red data points represent the beam angle deviations from the nominal set points during the delay and energy scan without any feedback stabilization. The blue data points, show the pointing error while the feedback control system is actively correcting for the deviations from the set-point. The black line is the moving median of 180 measurement points. The spread around the median is the shot-to-shot jitter of the X-ray beam source, and up-stream optical elements. From the data in Fig. 3 two important quantities can be analyzed, the shot-to-shot variance and the peak-to-peak variation of the moving median. The first is a diagnostic, to evaluate whether angular vibrations from the stages during the coordinated movement have been imprinted onto the X-ray beam or if the feedback system introduces errors. The latter represents the absolute deviation from the nominal position and measures the misalignment.

**Fig. 4:**
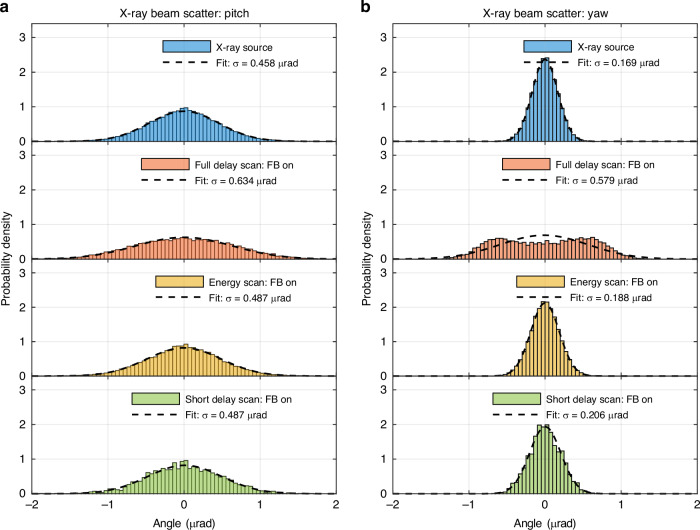
Analysis of the shot-to-shot jitter of the X-ray beam angle. Shown is the pointing data for static optics (blue), and during the delay scan (red), the energy scan (yellow), and a short delay scan with feedback in 4 degrees of freedom (green). The moving median shown in Fig. 3 was subtracted from the data so that the remaining variance corresponds to the shot-to-shot beam jitter. Panel (**a**) shows the beam pitch and panel (**b**). shows the yaw of the beam. Comparing the delay and energy scans with the reference data in blue, the feedback control marginally introduces detectable jitter, except in the case of the full delay scan. A splitting of the data points can be seen most clearly in the yaw and results from the systematic error caused by spurious interference

To analyze the shot-to-shot jitter, the moving median has been removed from the data points and the results for the data sets during active feedback are shown in Fig. 4b–d. Figure 4a shows the shot-to-shot jitter of the X-ray beam source in the static case. All results are also listed for comparison in Table 2a) for pitch and Table 2b) for yaw. For the short delay and energy scan the jitter is equivalent to the static case and the shot-to-shot jitter during the open loop scan. However, the long delay scan shows a splitting distribution, best understood from the histogram in Fig. 4b. This is a systematic artifact introduced by spurious interferometers due to changes in absolute length of the interferometer^30^. It could be minimized by an alignment on the diode to balance the effect equally on all four quadrants, however during this experimental time, the interferometer accumulated an alignment offset and the error became apparent. Remote balancing is an option to reduce the error in future iterations of the experiment and can be removed on the length measurement with balanced detection^31^.

**Table 2:** a Measured errors during the energy and delay scans, as well as the static reference for the beam pitch of the X-ray beam. b Measured errors during the energy and delay scans, as well as the static reference for the beam yaw of the X-ray beam

a						
Pitch error	Jitter: sigma (fit 95% CI) (μrad)		RMS (μrad)		Drift: moving median peak-peak (μrad)	
Feedback	Off	On	Off	On	Off	On
Static reference	0.46 (±0.01)		0.54		0.79	
Short delay scan	0.44 (±0.03)	0.49 (±0.02)	1.88	0.66	2.94	0.28
Full delay scan	0.46 (±0.02)	0.63 (±0.01)	1.27	0.66	4.22	0.68
Energy scan	0.49 (±0.03)	0.49 (±0.01)	1.60	0.86	4.07	1.26

b						
Yaw error	Jitter: sigma (fit 95% CI) (μrad)		RMS (μrad)		Drift: moving median peak-peak (μrad)	
Feedback	Off	On	Off	On	Off	On
Static reference	0.17 (±0.00)		0.33		0.18	
Short delay scan	0.20 (±0.01)	0.21 (±0.01)	1.93	0.24	5.39	0.16
Full delay scan	0.22 (±0.01)	0.58 (±0.01)	4.98	0.67	10.34	1.22
Energy scan	0.37 (±0.02)	0.19 (±0.00)	4.23	0.88	18.75	1.78

The feedback control system reduced the peak-to-peak drift of the moving median in all three scans. The alignment drift during the long delay scan was reduced from 4.22 μrad to 0.68 μrad in pitch and from 10.34 μrad to 1.22 μrad in yaw. During the open-loop energy scan, the motion of the optics deviated so far from the programmed nominal trajectory that the Darwin width of the crystals was not wide enough for the photon energy, and the Bragg condition for the reflection for the X-ray beam was lost after an energy change of 4.5 eV. With the closed feedback loop, we could extend the energy scan to 10 eV and minimize the drift to 1.26 μrad and 1.78 μrad for pitch and yaw, respectively. The short delay scan shows exceptional stability comparable to that of the static case.

The short delay scan stability is a result of a slightly different controls diagram implementation. Instead of correcting the DWS signal with the DPS signal to compensate for pitch and yaw, the DPS signal itself was used in a feedback loop to control M1 pitch and yaw. This resulted in a stable output beam in tilt and displacement with a peak-to-peak median drift of 0.234 μrad in pitch and 0.15 μrad in yaw. The feedback degrees of freedom were not well diagonalized, which leads to a Bragg condition misalignment of M1 and M2 beyond the 2.5 ps range. The result, however, demonstrated the potential that with feedback in 5 degrees of freedom, the output beam can be stabilized to an equivalent drift of 100–150 nrad per optic.

## Discussion

We have demonstrated dynamic stabilization of X-ray beam trajectories to better than 0.25 μrad peak-to-peak angular error for a length change equivalent to 2.5 ps time delay. This is required to align and operate an X-ray regenerative amplifier, as described in ref. ^14^. They present the requirements of any individual RMS optic drift to be below 0.35 μrad, which is equivalent to 0.6 μrad for three optics. The first step in cavity alignment is to close the X-ray beam trajectory and hence establish the transverse alignment. A single X-ray pulse will be circulated and by aligning it to the input and output apertures of the undulator it will be following the same beam path as the electron beam^32^. Afterwards the high repetition rate electron bunch train will be turned on and injected into the undulator. Then the length of the cavity is adjusted with a delay scan to find the temporal overlap by matching the cavity length to the bunch separation time. The starting length is determined by conventional position measurements and has an error of 10^−6^, or ±1 ps, assuming a 1 μs bunch spacing at 1 MHz repetition rate of the accelerator. Our interferometric system allows for precise length adjustments of the remaining ±1 ps while maintaining the transverse alignment within the specifications.

For our analysis, we distinguish two timescales for vibrations. Jitter, which happens on short timescales, is faster than hundreds of microseconds, and drifts, happening on the slower side. In our experiments, the motors were moving the optics in a way that the energy or delay of the half-cavity would change while preserving the pointing of the reflected X-ray beam. We observed that the measured jitter, while the motors were static, was due to the pointing jitter of the X-ray beam originated upstream, and it did not significantly increase during the motor movement, see Table 2.

Longer delay scans and energy scans show a significant reduction in peak-to-peak drifts with a 3-degree of freedom feedback control system architecture as compared to the open loop scans. Operating with a 5-degree of freedom feedback loop system architecture was not successful as the feedback actuation was not diagonalized to the 5-axis basis, and hence the feedback to M1 and M2 led to diverging angular corrections. The two Bragg crystals became misaligned from each other, while the output beam stabilization was excellent. A better mechanical separation of angle and displacement actuation as well as more time to diagonalize it will resolve this in future iterations. The achieved output pointing error of 1–2 μrad peak-to-peak drift is already close to what would be required for realizing a high-gain cavity system, as the photon-to-electron interaction in the undulator leads to a strong optical guiding and thus reduces the tolerable RMS mirror error to 0.5 μrad per optic^14^.

Another result of our analysis is that relatively simple stepper motor-driven motion stages did not add any measurable short-time jitter beyond the natural pointing jitter of the FEL with self-amplified spontaneous emission (SASE). The active position control thus only must compensate for the movement caused by the mechanical play and systematic movement errors of the motion stages. The requirements on the response time of the mechanical stages are uncritical and allow actuators with millisecond response times in future realizations.

The performance of the interferometric measurement has been compromised during the experiment, most likely by spurious reflections. Mitigating these parasitic beams is a first step, but even if this cannot be achieved, the measurement can be made insensitive against it by balanced detection of two photodetectors for the length measurement, and with beam position alignment to the QPD for the DWS measurement. A five-axis control architecture also resolves it. There is a clear path to extend the performance as demonstrated in the short delay scan to larger range and more complex adjustments ranges by extending the actuation degrees of freedom to 5 axis and mitigate stray light sources. The low gain X-ray FEL oscillator requirement is within reach with the presented methodology.

We envision this system as an effective motion tracking and error compensation mechanism for a variety of X-ray tools such as monochromators, delay lines, and mirror systems. For realizing a large-scale cavity as described in Fig. 1a), the motion guiding system can be expanded to include two ‘local’ tracking systems to secure motion accuracy of the components within individual optical bench on each end. Another single beam ‘distance + angle’ interferometer can be used to connect the two optical benches separated by large distances to make the two benches virtually ‘monolithic and stable’. Such an architecture utilizing relatively mature optical interferometer methodology will be essential in realizing future cavity-based X-ray free electron lasers driven by high repetition rate accelerators.

## Materials and methods

The following section describes the details of experimental set up, data acquisition system, and data processing protocol.

The experiment was performed at the X-ray Pump Probe (XPP)^29^ instrument at LCLS. The photon energy of the FEL was calibrated near the absorption edge of Ni K shell and set to 8.34 keV, with a bandwidth of ~30 eV full width. A float zone Si(400) crystal and a synthetic single crystal diamond (400) crystal were chosen as the Bragg optics. The grazing incidence mirror has a length of 300 mm and was coated with Pt. The cavity was initially aligned with monochromatic beam at 8.350 keV for calibration^11^. At this photon energy the Bragg angle of the Si crystal M1 is 33.14°; the diamond crystal M2 has a Bragg angle of 56.36°. The starting angle of the grazing incidence mirror M3 is at 0.5°. From this starting point the energy scans were executed in the negative direction from 8.35 to 8.34 keV.

The Bragg crystals are mounted onto three-axis piezo mirror mounts (Newport PSM2SG-D) that hosts both the X-ray crystal and the mirror for the interferometer IR laser beam. The piezo motors are mounted on stepper motor driven goniometer stage stack with larger range rotational motions to steer the beam reflection in both horizontal (yaw, *θ*_1,2_) and vertical (pitch, *χ*_1,2_). In addition, M1 can translate in in z direction (z_1_) along the incoming beam direction. M2 can translate in both the beam direction (z_2_) and the transverse horizontal direction (x_2_). The grazing incident mirror M3 has stepper motor driven motion to adjust the incidence angle (*θ*_3_) and translate in transverse horizontal direction (x_3_). Pre-programmed motion trajectory executing an Energy and Delay scan have the following motion vectors:

The laser interferometer is routed parallel but above the X-ray beam, at an elevation 25 mm higher. It’s in plane angle closely follows that of the X-ray except a higher grazing angle was used on the grazing incidence X-ray mirror M3 by 0.5 to reduce clipping by the mirror edges. The optical mirrors and X-ray crystals on M1/M2 motion stack were rigidly connected mechanically to minimize angular relative drift. The layout of the laser interferometer is shown in Fig. 3a. It is in a heterodyne Mach–Zehnder configuration operating at wavelength of $\lambda =1064\,{\rm{nm}}$. The optical beams are in-coupled into the vacuum vessel via polarization preserving optical fibers and couplers. For the heterodyne interferometry the laser light from one fiber has a frequency offset of 4096 Hz as compared to the light from the other fiber. When the two beams are merged and interfered with one another, a beat note at the heterodyne frequency is observed. A single element diode is used to measure the length change $\Delta l$ of the interferometer from the phase change $\Delta \varphi$ of the interferometric beat note as

$$\Delta l=\frac{{\rm{\lambda }}}{2{\rm{\pi }}}\cdot \Delta {\rm{\varphi}}$$

The data processing in the CDS is illustrated in Fig. 2b and based on the digital two quadrature demodulation described in ref. ^33^.

The beam delivery via two optical fibers can introduce differential phase noise into the measurement system, which was tracked and compensated. In Fig. 2a the optical layout of the laser measurement system is shown. The yellow and blue areas highlight the detection of the two interferometers, in yellow the reference interferometer and in blue the measurement interferometer. The reference interferometer is used to measure phase noise of the laser preparation and delivery system. This measurement is subtracted from the measurement interferometer readout to remove common mode phase noise. In addition, a fiber stretcher is used to cancel the differential path length noise in a feedback control loop. The phase noise and therefore length measurement is fully differential and does not track absolute path lengths.

The beam pointing deviation is measured via DWS and DPS with a QPD. DPS is the normalized position measurement on a segmented photodiode and measured as

$${{\rm{DPS}}}_{x,y}=\frac{{P}_{\text{right},\text{top}}-{P}_{\text{left},\text{bottom}}}{\sum \,P}$$

Calibration to position in $x$ and $y$ on the photodiode is related to the beam size ${\omega }_{{\rm{m}}}$ of the measurement beam on the diode as well as the power ${P}_{{\rm{m}},{\rm{r}}}$ of the measurement and reference beam respectively. Following ref. ^34^, the position on the diode is measured to be

$$x=\sqrt{\frac{{\rm{\pi }}}{8}}\cdot {{\rm{\omega }}}_{{\rm{m}}}\cdot \frac{{P}_{{\rm{m}}}+{P}_{{\rm{r}}}}{{P}_{{\rm{m}}}}\cdot {{\rm{DPS}}}_{x}$$

$$y=\sqrt{\frac{{\rm{\pi }}}{8}}\cdot {{\rm{\omega }}}_{{\rm{m}}}\cdot \frac{{P}_{{\rm{m}}}+{P}_{{\rm{r}}}}{{P}_{{\rm{m}}}}\cdot {{\rm{DPS}}}_{y}$$

The calibration factor is

$${c}_{{\rm{DPS}}}=\sqrt{\frac{{\rm{\pi }}}{8}}\cdot {{\rm{\omega }}}_{{\rm{m}}}\cdot \frac{{P}_{{\rm{m}}}+{P}_{{\rm{r}}}}{{P}_{{\rm{m}}}}$$

The DWS signal is measured in relation to the interferometric phase measurement ${\rm{\varphi }}$ as

$${{\rm{DWS}}}_{\varphi ,{\rm{yaw}},{\rm{pitch}}}={1/2{\rm{\varphi }}}_{\text{right},\text{top}}-1/2{{\rm{\varphi }}}_{\text{left},\text{bottom}}$$

Following ref. ^28^, the tilt ${\rm{\alpha }}$ of the wavefront is related to the DWS measurement in yaw as

$${{\rm{DWS}}}_{\varphi ,{\rm{yaw}}}\left({\rm{\alpha }},x\right)=\sqrt{\frac{2}{{\rm{\pi }}}}\cdot k\cdot {{\rm{\omega }}}_{\text{eff}}\cdot \left({\rm{\alpha }}-\frac{x}{{R}_{{\rm{m}}}}\right)\cdot F\left({\rm{\sigma }}\right)+O\left({{\rm{\alpha }}}^{2},{x}^{2}\right)$$

With the wavenumber $k$, the effective beam radius ${\omega }_{\text{eff}}$, the beam tilt against the reference beam $\alpha$, the horizontal displacement on the QPD $x$, the radius of curvature of the wavefront of the measurement beam ${R}_{{\rm{m}}}$, and

$$F\left({\rm{\sigma }}\right)=\frac{1}{\sqrt{2}}\sqrt{\frac{1+\sqrt{1+{{\rm{\sigma }}}^{2}}}{1+{{\rm{\sigma }}}^{2}}},{\rm{\sigma }}=\frac{k{{\rm{\omega }}}_{\text{eff}}^{2}}{4{R}_{\text{rel}}},\frac{1}{{R}_{\text{rel}}}=\frac{1}{{R}_{\text{r}}}-\frac{1}{{R}_{\text{m}}},\frac{1}{{{\rm{\omega }}}_{\text{eff}}^{2}}=\frac{1}{{{\rm{\omega }}}_{\text{r}}^{2}}-\frac{1}{{{\rm{\omega }}}_{\text{m}}^{2}}$$

${R}_{\text{r},\text{m}}$ and ${{\rm{\omega }}}_{\text{r},\text{m}}$ are the radius of curvature and beam radius at the QPD of the reference and measurement beam respectively. The equivalent equation for pitch and vertical displacement holds true. The DWS signal can be corrected with the DPS signal to turn it into a pure angular measurement. We calibrated the DPS correction signal by displacing the laser beam on the diode by $x$ in the horizontal direction, without introducing a tilt ${\rm{\alpha }}$, which results in

$${{\rm{DWS}}}_{\varphi ,{\rm{yaw}}}\left(x\right)=-\sqrt{\frac{2}{{\rm{\pi }}}}\cdot k\cdot {{\rm{\omega }}}_{\text{eff}}\cdot \frac{x}{{R}_{m}}\cdot F\left({\rm{\sigma }}\right)$$

This is subtracted and the remaining DWS signal only depends on the angle $\alpha$

$${{\rm{DWS}}}_{\varphi ,{\rm{yaw}}}\left({\rm{\alpha }}\right)=\sqrt{\frac{2}{{\rm{\pi }}}}\cdot k\cdot {{\rm{\omega }}}_{\text{eff}}\cdot {\rm{\alpha }}\cdot F\left({\rm{\sigma }}\right)$$

The angle is measured as

$${\rm{\alpha }}=\sqrt{\frac{\pi }{2}}\cdot \frac{1}{{k\cdot {\rm{\omega }}}_{\text{eff}}\cdot F\left({\rm{\sigma }}\right)}\cdot {{\rm{DWS}}}_{\varphi ,{\rm{yaw}}}$$

In our signal processing chain, the phase is converted to an equivalent length change before the DWS signal is calculated, hence ${k\cdot {\rm{DWS}}}_{l,{\rm{yaw}},{\rm{pitch}}}={{\rm{DWS}}}_{\varphi ,{\rm{yaw}},{\rm{pitch}}}$. The angle measurement in our signal processing chain is calculated as

$${\rm{\alpha }}=\sqrt{\frac{\pi }{2}}\cdot \frac{1}{{{\rm{\omega }}}_{\text{eff}}\cdot F\left({\rm{\sigma }}\right)}\cdot {{\rm{DWS}}}_{l,{\rm{yaw}}}$$

$${\rm{\beta }}=\sqrt{\frac{\pi }{2}}\cdot \frac{1}{{{\rm{\omega }}}_{\text{eff}}\cdot F\left({\rm{\sigma }}\right)}\cdot {{\rm{DWS}}}_{l,{\rm{pitch}}}$$

The calibration factor is

$${c}_{{\rm{DWS}},l}=\sqrt{\frac{\pi }{2}}\cdot \frac{1}{{{\rm{\omega }}}_{\text{eff}}\cdot F\left({\rm{\sigma }}\right)}$$

All calibration factors have been determined experimentally by rotation and translation of the motion stages.

The beam tilts $\Delta \alpha$ and $\Delta \beta$ and length change $\Delta l$ of the interferometer are related to the coordinate system of the half-cavity experiment, with the mirror tilts M2 yaw ($\Delta \theta$) and pitch($\Delta \chi$) and the cavity length $\Delta z$ as follows:

$$\Delta z=1/2\cdot \Delta l$$

$$\Delta \theta =1/2\cdot \Delta \alpha$$

$$\Delta \chi =1/2\cdot \Delta \beta$$

The data is presented in the coordinate of beam pitch ($\Delta \beta$) and yaw ($\Delta \alpha$) projected to mirror M2 and interferometer length change ($\Delta l$). The X-ray diagnostics does not allow us to measure the length, only the beam tilts.

The data processing is divided into two independent data acquisition and control systems. The LCLS data acquisition (DAQ) system records the data stream related to the X-ray diagnostics, the accelerator condition, as well as the beamline status. It is event-driven, synchronized to the generation of X-ray pulses on a 120 Hz clock. The images from the X-ray camera are captured at this rate. Post processing reduces the data to smaller packets containing fitted beam x-centroid and y-centroid, in synchronized array format with other beamline data such as pulse intensities, motor positions, accelerator parameters, etc. The synchronous motion control was executed using built in functions of the Aerotech Ensemble multi-axis motion controller. An analog signal linear to position within the planned motion trajectory was generated and sent from the controller to the analog input of the LCLS DAQ. This synchronized ADC value can then be used to calculate the equivalent energy change and time delay of a cavity, for each recorded X-ray beam image. Independent of the LCLS DAQ, a second data acquisition stream runs in parallel via the LIGO CDS and is responsible for the interferometer data processing and the real-time control loops. The sampling rate is 65536 Hz, but the data is filtered and stored as proprietary frame files at a rate of 2048 Hz. This data acquisition is not synchronized to the LCLS data acquisition system, but to the network timing protocol (NTP) and therefore to GPS time. Software tools allow quick and easy access to stored and live data. We have exported selected frame data to mat files.

The X-ray and interferometer data were then analyzed in MATLAB. As part of the X-ray data set, the intensity on the detector was recorded and a threshold was used for the X-ray data to exclude weak X-ray images without sufficient signal-to-noise ratio for an accurate centroid position. The centroid data is converted to an equivalent tilt angle of the beam. To measure shot-to-shot variance over the course of the experiment, a moving median of 180 data points was calculated and subtracted from the raw data. Large-scale movements are suppressed and the short-term jitter of the X-ray beam on the detector is shown in the histograms in Fig. 4, normalized to the probability density function (pdf). A fit to the normal distribution gives the standard deviation and is shown in Table 2. For the calculation of the shot-to-shot variance of the open-loop energy scan, data points had to be excluded because there were not enough data points available to calculate the moving median above 4.5 eV.

## Data Availability

Data sets generated during the current study are available from the corresponding author on reasonable request.
